# Supplementary material for: Synthetic CpG islands reveal DNA sequence determinants of chromatin structure
Source: eLife. 2014 Sep 26;3:e03397. doi: 10.7554/eLife.03397 (PMC4204011; doi:10.7554/eLife.03397)
Supplement: Figure 1—source data 1. — Table showing properties of constructs used in these studies, including length, base composition and CpG frequency (observed over expected CpG ratio; o/e) of all tested constructs. Note that the o/e ratio takes the overall G + C content into account and therefore the High CpG/ Low G + C sequences have a high o/e ratio. DOI: http://dx.doi.org/10.7554/eLife.03397.004 [file elife03397s001.docx]

|  | **Length in bp** | **G+C content** | **A+T content** | **Nr of CpGs** | **o/e CpG ratio** | **CpGs/100bp** |
| --- | --- | --- | --- | --- | --- | --- |
| **PuroGFP** | 1267 bp | 66.5% | 33.5% | 140 CpGs | 1.0 | 11 |
| **Artificial CGI 1** | 1055 bp | 69.5% | 30.5% | 127 CpGs | 1.0 | 12 |
| **Artificial CGI 2** | 1014 bp | 64.8% | 35.2% | 85 CpGs | 0.8 | 8 |
| **Low CpG/ High G+C** | 1008 bp | 64.4% | 35.6% | 10 CpGs | 0.1 | 1 |
| **High CpG/ Low G+C** | 1007 bp | 40.3% | 59.7% | 100 CpGs | 2.5 | 10 |
| **High CpG/ Low G+C 2** | 1096 bp | 41.8% | 58.2% | 105 CpGs | 2.1 | 10 |
| **High CpG/ Low G+C 3** | 1002 bp | 40% | 60% | 84 CpGs | 2.1 | 8 |
| **High CpG/ Medium G+C** | 1006 bp | 51.6% | 48.4% | 100 CpGs | 1.6 | 10 |

Wachter et al 2014 Figure 1-source data 1
