# Supplementary material for: Synthetic CpG islands reveal DNA sequence determinants of chromatin structure
Source: eLife. 2014 Sep 26;3:e03397. doi: 10.7554/eLife.03397 (PMC4204011; doi:10.7554/eLife.03397)
Supplement: Supplementary file 1. — DOI: http://dx.doi.org/10.7554/eLife.03397.014 [file elife03397s002.docx]

***Wachter et al – Supplementary File 1***

**CGI-like DNA sequences**

Puro GFP

AGCTTGTCACCGAGCTGCAAGAACTCTTCCTCACGCGCGTCGGGCTCGACATCGGCAAGGTGTGGGTCGCGGACGACGGCGCCGCGGTGGCGGTCTGGACCACGCCGGAGAGCGTCGAAGCGGGGGCGGTGTTCGCCGAGATCGGCCCGCGCATGGCCGAGTTGAGCGGTTCCCGGCTGGCCGCGCAGCAACAGATGGAAGGCCTCCTGGCGCCGCACCGGCCCAAGGAGCCCGCGTGGTTCCTGGCCACCGTCGGCGTCTCGCCCGACCACCAGGGCAAGGGTCTGGGCAGCGCCGTCGTGCTCCCCGGAGTGGAGGCGGCCGAGCGCGCCGGGGTGCCCGCCTTCCTGGAGACCTCCGCGCCCCGCAACCTCCCCTTCTACGAGCGGCTCGGCTTCACCGTCACCGCCGACGTCGAGGTGCCCGAAGGACCGCGCACCTGGTGCATGACCCGCAAGCCCGGTGCCTGACGCCCGCCCCACGACCCGCAGCGCCCGACCGAAAGGAGCGCACGACCCCATGGCTCCGACCGAAGCCACCCGGATCCACCGGTCGCCACCATGGTGAGCAAGGGCGAGGAGCTGTTCACCGGGGTGGTGCCCATCCTGGTCGAGCTGGACGGCGACGTAAACGGCCACAAGTTCAGCGTGTCCGGCGAGGGCGAGGGCGATGCCACCTACGGCAAGCTGACCCTGAAGTTCATCTGCACCACCGGCAAGCTGCCCGTGCCCTGGCCCACCCTCGTGACCACCCTGACCTACGGCGTGCAGTGCTTCAGCCGCTACCCCGACCACATGAAGCAGCACGACTTCTTCAAGTCCGCCATGCCCGAAGGCTACGTCCAGGAGCGCACCATCTTCTTCAAGGACGACGGCAACTACAAGACCCGCGCCGAGGTGAAGTTCGAGGGCGACACCCTGGTGAACCGCATCGAGCTGAAGGGCATCGACTTCAAGGAGGACGGCAACATCCTGGGGCACAAGCTGGAGTACAACTACAACAGCCACAACGTCTATATCATGGCCGACAAGCAGAAGAACGGCATCAAGGTGAACTTCAAGATCCGCCACAACATCGAGGACGGCAGCGTGCAGCTCGCCGACCACTACCAGCAGAACACCCCCATCGGCGACGGCCCCGTGCTGCTGCCCGACAACCACTACCTGAGCACCCAGTCCGCCCTGAGCAAAGACCCCAACGAGAAGCGCGATCACATGGTCCTGCTGGAGTTCGTGACCGCCGCCGGGATCACTCTCGGCATGGACGAGCTGTACAAGTAA

Artificial CGI 1

AGCTTCTAGCACAGGAGTTGACTCGGCAGGTGCCGTAGTGCGGCTTGGCAGCGGGACGTCGCTGGTCGGGCCTGACTGGGCGCTACGCCGGTTGTGGTCACCTGAACCGCATCTGGGCCGTCGCTCGCTTCGCGGGCTCTGCAGCCGGACTCCACCAGCGGGACCTCACACGCTCGGGTGAGCCGTCCTAGGCCGCTTGCGCCAACCCACGGGGTAGGCCTGGCGAGACGCACGGGCAGTGCCGTTCCTGAGGTCCGCGGTGCTCCCTGCCCAGACGCCTAAGCACGCTCCACCCGTGCCTCGGGTTCCGGGGATTTAGGCCGCACTGTGCGCCATTCCGCGGCTCGCAGCCACCGAAGTGCCGCGTTCCCTCCTACCCTAGCCGCAGCAGCGTCCAGGGCCAAGAGGCTCGGCTACGGTCCGACTTTTGCACTGGGCAGTCGCAGCGTCGTCCTCTCGCGACGCGGGTGCAGAGGTCGGCGGGTCGTAACCGACTGCCAACCAGGCGCAGGCTGGTACCCACCGGGTGGGCATAACGTCCATCGCCTGGGAGGTGGGACGCGGACTCGTCGGACACGTCGCCTCTGCGTGTGTCGCGGGCCAGCAGCCAGCCGGCCTGGATCTAGACCCGGCGGCCATTGCAGCGAAGGGAGGTTCGCCACGGCGTAGCCCAGTCGCGCGTGTACCCAGCGCATCCGCCGGAGCCCATCCCCGCTCACGGTCGAAGCCCACCAGGGTGTCTCCACCGCGGCCTTTGCCCACCTCGCTGGTCCACAGCCGAGCGGTCCGCTCTGACGCAGCGTCCTGCCAGCCCTCGACGGCCGACCCCTGACTAGTTGGACCGCCTCCAGCGGCTGCGGGACACAGCTCCGGCGGTGGGGACGCACCGAATGGGACACCGGCACTCCTAGGGCGTACAGCTGCTCGCAACGCAACCGAAGGCAGCCTCCTACTGGCGGGCCGATCCGGCCTACATGGCCCTGGCCCATCATCCCGCGCAAGGTCGCACAATCGACGTGCACGACGGCAGCACGCCAGGTGACGTTCCCGGAGCA

Artificial CGI 2

CTCACTATAGGGGCTGTTCCTGCCACGAGGCGTAGCACACAGGGAGACGGTCACGGGCGCGGTCCAGACGCGCGGCCCGGTGCCCCCTGTCCGAACAGGCCAGTTGTGCAAGCCGACTGAACTCGCCCCATGACGTGTGTGGCTGACCACGCGCACCAGAGTAGGCCCCAACGCGACGATCCCACGCCGCGCACCTCGGCTTCGGCGTGGGCGTTCCCGCACCTACCAAGGTGGCATCTGGAGCACGTATAGTAGCGCAGGCCTCCGCTCTGGCCAGCCTGCGCATAGCCAGGCTAGAGGGAGAGAACTCACGCCACGGTCGATCCTGGTCCCGTGCACCCCCGTAGCCTTCCTGCGCCACAGTGACAAGTTCACCCGCTGGGCGTTCTGAGTGGACGGAGCATGTGGCTGGGCCCGGACGGGCCCGAAGGGGGGCCTTGCATAGGCTGATTGCCTGTGGCTGTAGTCTCGTTCCCTTGCTCTCCGCTCCACCCTGATCCAGATAACGCCCATCCGGTCCCGCACAACTCGGCCTACTCTGATACGTGTCCAGAGGCCGTCCCGAAGGCAGTGGGAAGTAGTATGGAGGCGTGTGACGATGCCGCACAGGGAATGCCTCCTCTCGGGAAGAGTTGCTACCGAGAACCAGTGCGCCCGTCCCGCACGGCTCCTTGTGCCGGGAACCCTCCAGGGAGCGCTGAACGCTTGCCGAAGAAGCTGCGCGGTCTGGGCTAAGAAAGCTCACCTGCCCTCTCGCTCGTTCATGAGCCCAGACAGCCCATGCCGGACTCCGGGCCTGCTGCGCAGGACGGCTGTGCTTCACATTGGTTACCCAGCCTGAGCGAGTGGCAGCCACAAGATGCTACTGGAGGACGGAGTTGTTCACCCTAAGGGGGATCGCCGAGCCTTGGGTCCGTCCCGGACCCCTGGTAGCCTCAACTCCTCAGCACCTACTAGGCCACGGCCCGTACTTAACGGTCTGATCCGGCCCCGCTATGCTTACCCCTGCGTCCG

Low CpG High G+C

GCTAAGCTTGGCCCCTGGGGGAAGCCCCACATGCCTGGGACCCCAAGCTGGACTTCCGTCATGCAAGAGTACCAGGTACAGGGGACCCCTTAGTGTCTCCCTGGCAAGTGCCCCCAGGGGACCAGGGTGGGCATCCCCCCACCTAAGTGGCCACCCCTCCCTCCACCACTGTCCCCTGAAGGACATGTTGAGCCTGCCTCACCGGGGTGTGGTGGCCACTGGGTCTCCAGGGCTTGCCAAGGGGTCAGAATACTTCCGGGGTGTCTACCCATCCCCACCCTAGGGGATGCTAAGCGGGGGTCCAGCCCTGCCCATTCCCCCAGGGGTAGGAGGGGGTCCCTGGTGCCCCTCCCCAGAAAGGAGGCCAGGGGAGTGGGGGGAGCCTAAACTAAACCCACTCAGCCCCTGGGCCCAGTTGGGAACAGATATGGCTAGGCGGGGAGGCAGGGGGCTGATGAGGGGCCTGGTAAGGTCTCCCTCCCAAGTGGGGGGGTGGATGGGCCCAGAATCCTATGAGGACACATATCTTTGACTGGGCAGCTTCAGAGGGGTAGGGCCCTTGAGGGCTGGGTAGAGTCCCAAGGCCCCAGGGGGGATGTGACCCCCCTATGTCCTCAGCCCCCCCAGACCACAGAGAGTTCAGGAAGGAGGGTAGCCCCGCCTCTCCAGGGCAGGTGACCCAGGGCCCCCTGGTAACTGGGGGGGGAGCACCCTCATTGAACCCCCCGAGCCCATGTCAGGTGGCAGCCACTCCCAGCCAGAAGCCCTGAGGGCCCATCCCAGGTGGCCCCTAAGGGGGAGGGGGGATTCCCAGGAATATTCTCCCAGCTTCAGGGCCTCAGTGAGAATCATGAGGGGCCCTGGCTCCCGCCATACCCCACCAGCATATGGCCTCTCCCCGGGTTCAGGGAGAAACCCAGTGGGGCAGCAAATTAAGCATCTCCCACTGACCAGAGCATTGGAGGTAGGGGCTTCTGTAGGATGCCGGCCAAAGCTGCCAGCTGAACCCCTGAATTCTC

High CpG Low G+C 1

CGTAAGCTTCTATAGCACACGGGCCAACGACAACGCTGGGCGATTTAACGTTTAATGTCGTATGAGTCTCGATAGCGAGGTTGGCACTCCGACCAAAAACCGAACATTGAATCTAACGGAATACTGTCACGTTAATAACGTATAACGAAATATACGTATTTTAACTATGAACGCATATAACGATTATCGCAGAACAAATTTTACGAATCAATAAAAAACGTGATACGTAACTCGTTCGTTCAGAAGATTATGCGGACGAAAGAATGTACGAACTCGTTGTATTTCTTGCGCGATATACGTAGTACGTGTTTCGTATGTAGTACCGGAAAGTATCGAATCATTTCGATCGTACCTCACCGTTTCGACAACTACGATACGCTAACAGTTGTTCGGCTATACAGCTTACGTCGCACAGTAGACGATTCCGACATGACGCAACTTATCGAAACAATTTCGATTTTAACGACGTACAAAATCGATTCGAATCACACTCGATATCGTACTATACAAATGTGAAATTCGGTCTCACTTTCTTGATCCGTTTAGCGAATCTCTTTAACGCTTCGAGATTTAGTAGTTTTCGATGTAAATTTGACGAAGTTTGTCGCGCATGAAAGAGTAAACGTCAATCTTCTCGATCTTATAACTATCGACCGAGCCGCGCCTTAGCTTCGCATATATGACAAATGACAAAACAAACGTGATTGTCGCAGAATACGTTCTTTGACGTCATTTTAGACGAAATCGACTCGACTTCAATACGTTATACGATACGAATGTTGATCTGTTCGTGTACGCTTCGATCATTACTCGATCGGGTAGTTGTGCGTTTTCGCATTGCGCACGACGAATTATCTGTAAACGTTTCCGGGGGATGCGACCTCTCAGATCGTTATAAATGCTATTAACGTTATTATTCTTCCTTGGATAAGTCAAGTTCGAACGTGCTAAGAAGTGTGTTACTATATTACGAATGAACGAGAGAGCATTAATACGACGATTCCCTAAATGAATTC

High CpG Low G+C 2

CGAATTGGCGGAAGGCCGTCAAGGCCACGTGTCTTGTCCAGAGCTCAAGCTTAATACGATCAACTACATTCGATCTCAGTTATATGAAACAGATAGTATACACATTTTCTACAGGCCTAAACTTACGGACGATCAGATTTGTACTCATTGTTGTGTAATCGGTACGCTCTTCGCTCTACTTGTAACTATCGTTCTCTATTGTCGGACTAAACTATGTTCTCAACGATCAAAGTACGTTAGACTTGACGTAGTAGAATCGTAATTTAAGACACGGCAGATGCGACGACAATAATGTCTAGAGACGAACGCTACTTCACGTATCAAGTACAACGATCACTAAATCGTTAGATCATTACGTGATATCGATTGTTCGTTCTGCGATGTACGATTTGAAAGATTCGATTGTGACAACGATCTAACAGATCGGAGAGTTCGTAAGTTTGTCGAAATTTTCTTCGAGTGAACTATAAATCGCTACACTGACGTAAAGATGAGATCAATCGAGTCAATGAATTTAACGATTACGTTCAACGCTACCAACGTCACATTCAACGATACTAATCGGATCGGTATCGCTATCGCATACTTCGTTCATTCACGTAATATCGATTATATTGACGCAATTCGCGATCAGTCGAAGTCGCATATACGTTAATCGTAACATCGTTTTAAACGTCGATTTCTGTTCGTTTAGTACGAAGACACAATTCGAGATCGCGGAATCGTAAGTAAAACGGATTCATCGTGTTTCGCTACTCTTGTCGCTGTCAGACGGCTTCGAAATCGCTAAGTAGATCGACTTCACGCATAGAGAATACGCGACTACGTTCTTTCTCGTGTTTTACGCTGAACGGTCGTCAACTATTCGTCACGACATAACGCTAGACGGATTATCGTAAATTCACGAGGTCGCTTCACTACGTTGTCATCATCGACGATAACTCGTTTAGAAATGTTGACGGAGTGATCGAAAAGAACTATAACGATCAGTTAAGTATCGTATAATCGAAACGCACGTCTGAATCAATATCTGTAGAGTCTAGATCGACTCGACGGTAAGAATTCGGTACCTGGAGCACAAGACTGGCCTCATGGGCCTTCCGCTCACTGC

High CpG Low G+C 3

ACGTAACAAACGCTGATCTTACGTATGAGACGTCGATTGATGATTCGAAAGTACTCCGTGTTCGCGGATTTATAATTGAACTACTAAATTGAGCTTGACCGAGACATTTTTCTTTACTCTTTAAAGTATCGTGAACGCTGCGATGTACTTGGATTCATCGTGATTTATACAACGTTGAACTGTTGAATATTACGACGTCTGACAGATTGACGACAGAAGTGAATAATCAGTACGAATCCGACGCTCGGTGTAATCAGAAACGTCTCTGATCACATCGCACTATCGAATAACGTAATTTACGATTGGGGTCGCACTAGGAACGTTAATCGCTAACTACATATCGGGGAGTGCCCATGCACCTTCATAACATCTCATTGAAATCGCATGTACAATATCATACTATTCGATGTATATCGTATTATACGTCCTTCGCTCTCCGGCGATATCCTTGTGCTACGCGTTTACACGCGTCTATGGTTGAGGAATGTCGAACACTACCTCCTAATTGAGAATACCTACCTCTAGTCAACCTTCATAGAAATCTCTTATTCTCATTCTAGTCTTCGAACGTTAGTACGCTTCTTCAGTCTTCGCGATGTTTTTACGCTATTCGACGAAACGCTCGTTACGTCTAACTAACCGGACGCGCAACAATTGTAAGTACGTGTTTCAGAAACGAATCATTTAACGTTTCGTCTAATTTTACCACAGAGTGGAATTACTTCAAACTTGAGATCGAAATAAAATCTTCGAGTGTCTGATCTCGCGGAACCATATATAGACGTTTGGGTTCGCTATTCGTACTTTTTTCGATCTTCGGAGGTTTCGTAAAACGGGGCGTGAAACTTCTATACAATCGCCCTCACAATCGCATCGTATTGATTTAACGAGACACGAACATAGAAGAGTTCGACGAGTTAGAAAAAAATCTCAACGATCTCTATTGAATGTACGTTCATAATTATCGATTTCTAGTTATTAATCATTTTAGTGTTCGGAAAC

High CpG Medium G+C

TCGTATCCTATGCAGATCgCTCTGCAACATGAAATTTaCGTTCACGTTGGTACttGTGGtATACCGTTATGCGCTGAAAAGTCGTAAACtCTGCGGTTAACCACGGGCCTAGACGGTAAGGCCCAGCCGGATTACATTAACTTGAGATGCCATTTCACTGCATTGCGTGGTTACGCATGCCTTCACTTCGGGAGCaTGGAAACGGGCCAAtCTcGTGTTGtAGAAGACTCGGCaTTTAACTGTCtCAAAATCGTGGCTACGATCATACGGTAAAGGCTTGAGAAGTATGGcGCCTCTCACTTTCTTAACgTTCTATGcGTGGAGCACtACgCAGGCTTGCCTaCTTTGcGGGGGAAGAGGCTCGCTTACgAATCgAAAAcGGTAAcGTCGCGGACCCGTAGtGCGAGCTAtCgTAGTTCCCGACgCATACgTCGAGCCTTGcGACCTACTCTCGCAGGTATAACGATGttCGCTTTACTcGAACTTCTCgAATCGTCCGGGAAGACCGAACCCGTGCTAATcGAAACgCTAACCCgAAATAGTGCgCATTtCGGTCCATGGAAtCGGGTTCATTACgAGTtCAACgACTAGCgTGACGAAACgTTATTaTCgAcGCAGGTAGTGCGAATGCTGCGGAGGAACGGGGGGCGAACCACGAAATGAACGGTATGCCGCGAGATGCGTAACTACACGTGCGCTCTTGTcGTAATTTAGGGTACgCTGtCCGGCCTTGCAACGCATCCTCGACACtACTGcGTTAAACCtAAGCTaCACAACCGGCTGCGTGTACCACGACAAAGAAAtAAGTAGCATtCGCAACATCATCTCCGGGCGCTGGGTGCGCAGCATTCGCCAGACATaCACGTGTTCGGAGAGatTCCAGATTACGCCGGCTAACAGAAATTCTGaCACAtCGATATTCtaCTCGTCCGTAGCGACTACCTCAAGCGGTTTCACTAAGCTGtGtCACATGTCGTGCGGCGTGGCttCATGaaCGGCCATGCAC
