# Supplementary material for: Synthetic CpG islands reveal DNA sequence determinants of chromatin structure
Source: eLife. 2014 Sep 26;3:e03397. doi: 10.7554/eLife.03397 (PMC4204011; doi:10.7554/eLife.03397)
Supplement: Supplementary file 2. — DOI: http://dx.doi.org/10.7554/eLife.03397.015 [file elife03397s003.docx]

***Wachter et al – Supplementary File 2***

**Primers**

*ChIP primers*

| **Gene desert 1** | **Forward Primer** | **Reverse Primer** |
| --- | --- | --- |
| 1 | AGCAATGGCAGAATGAGAGG | TCAAACTGGACACAGGGACA |
| 2 | GCACTGGGTAGCCATTTCAG | TTCTTCGTTCTCCACGATCC |
| 3 | GGATCGTGGAGAACGAAGAA | TGTGTGCGTGTTTGTCTCTGT |
| 7 | TTATTACGCCATGGCTCCAG | CACAATTTGGGGCATGACAG |
| 8 | GCAGAGCATGCTGTTGTTGG | CCAGGTAGGTCTTTGGTGCTG |
| 9 | CCAGCTGCTGACAGGGAATC | TTGAATATGGCTTCTCTCTTTCCAC |
| **Gene desert 2** | **Forward Primer** | **Reverse Primer** |
| 1 | TGATGGTGGCATATTGCTTTC | CAATGCTCCATTTCCCTCTCA |
| 2 | GGAAGGGTCACTGTGTCAAGC | GAGGCTGACCTTTCTCCTCCA |
| 3 | TGACTCTCTGCCATGATGTGG | CAGGAAAGCTGTTCCCACGA |
| 4 | GCATTTCAGGCTGAGGTATTGG | TGCAACCTGATCTCCAGCCTA |
| 8 | CCATTCTTTCCTGCGGTCAG | CAGCAGGCACTGAAACATGC |
| 9 | GCCCATTTGGAATTGGCTCT | TGGCAAGTTCTGTGTTTGCAG |
| 10 | AGGCAGGGATGGGAAGATGT | TCTTGCCTTAGGAAGCAGTGA |
| 11 | TGGCAGTAAGTGACACCGCTATT | TGGCTCTGAGGAGAGAAAGTGG |
| **β-globin locus** | **Forward Primer** | **Reverse Primer** |
| 1 | tgaaccaaaggccttctctg | agccatgctttttagctgga |
| 2 | gctgccagctatcaagaagg | agactccaccctgagctgaa |
| 5 | gagtcctggggaagggtaag | atggtgtgaggtctggaagc |
| 6 | atggtgtgaggtctggaagc | caagtgtgcaaaagccagaa |
| 7 | tgtcctcatgcttcacttgc | taccttcagtggcagcacag |
| **PuroGFP** | **Forward Primer** | **Reverse Primer** |
| 4 | TCTGGACCACGCCGGAGA | CCAGGAGGCCTTCCATCTGT |
| 5 | AAGGGCGAGGAGCTGTTCA | CCGGTGGTGCAGATGAACTT |
| 6 | TCAAGATCCGCCACAACATC | ATGTGATCGCGCTTCTCGTT |
| **Artificial CGI 1** | **Forward Primer** | **Reverse Primer** |
| 5 | CAGGAGTTGACTCGGCAGGT | CCAGATGCGGTTCAGGTGAC |
| 6 | GCGTTCCCTCCTACCCTAGC | CCTGGTTGGCAGTCGGTTAC |
| 7 | GAAGCCCACCAGGGTGTCTC | GGAGGCGGTCCAACTAGTCA |
| **Artificial CGI 2** | **Forward Primer** | **Reverse Primer** |
| 4 | ACCTACCAAGGTGGCATCTG | GGCGTGAGTTCTCTCCCTCT |
| 5 | ACATTGGTTACCCAGCCTGA | CAACTCCGTCCTCCAGTAGC |
| **Low CpG / High G+C** | **Forward Primer** | **Reverse Primer** |
| 5 | AAGCTGGACTTCCGTCATGC | GGCAGGCTCAACATGTCCTT |
| 6 | GGCTGGGTAGAGTCCCAAGG | GGTCACCTGCCCTGGAGAG |
| 7 | CCACCAGCATATGGCCTCTC | GGCCGGCATCCTACAGAAG |
| **High CpG / Low G+C 1** | **Forward Primer** | **Reverse Primer** |
| 5 | CGATAGCGAGGTTGGCACTC | CGTCCGCATAATCTTCTGAACG |
| 6 | AAGTTTGTCGCGCATGAAAGA | TCTGCGACAATCACGTTTGTTT |
| 7 | TGTTCGTGTACGCTTCGATCAT | CGATCTGAGAGGTCGCATCC |
| **High CpG / Low G+C 2** | **Forward Primer** | **Reverse Primer** |
| 5 | GGCCTAAACTTACGGACGATCA | CGTTGAGAACATAGTTTAGTCCGACA |
| 6 | CGATTGTTCGTTCTGCGATG | TGTGACGTTGGTAGCGTTGAA |
| 7 | ACGCTGAACGGTCGTCAACT | TGATTCAGACGTGCGTTTCG |
| **High CpG / Low G+C 3** | **Forward Primer** | **Reverse Primer** |
| 4 | ACGCTGCGATGTACTTGGAT | GCGTCGGATTCGTACTGATTA |
| 5 | CGCGTCTATGGTTGAGGAAT | ATCGCGAAGACTGAAGAAGC |
| **High CpG / Medium G+C 3** | **Forward Primer** | **Reverse Primer** |
| 5 | CCGGAGATGATGTTGCGAAT | ACACGTGCGCTCTTGTCGTA |
| 6 | CGGCATACCGTTCATTTCGT | TCGAGCCTTGCGACCTACTC |
| 7 | GTTTCCATGCTCCCGAAGTG | GTAAGGCCCAGCCGGATTAC |
| **ChIP controls** | **Forward Primer** | **Reverse Primer** |
| Sox2 | AGGAGGAGAGCGCCTGTTTT | CGGAGATCTGGCGGAGAAT |
| GAPDH | TTCGCACCAGCATCCCTAGA | TCTTGTGCAGTGCCAGGTGA |
| Actin | cctaatacggcttttaacaccc | cctgaggatcactcagaacgg |
| Hox A9 | GACTGGAAGCTGCAAGGACTG | GGGCCCTGGGCAACTACTAT |
| Hox C8 | GCTCCCCAGACAGTCCCTTT | GGCTGGGAGACGAGTTCTGA |
| M15 (-) | GGGACGGGAACAAATGATGA | GACTGCCGTTCTTTGCTTGG |

*Bisulfite primers*

| **Artificial CGI 1** | TTGGTATTTATYGGGTGGGTATA | ATCCAACTAATCAAAAAT |
| --- | --- | --- |
| **Artificial CGI 2** | AAGGTAGTGGGAAGTAGTAT | AACRAAAAAACAAATAAACTTTC |
| **High CpG / Low G+C 1** | TGTGAAATTYGGTTTTATTTTTT | RTTTATTTTATCATTTATCAT |
| **High CpG / Low G+C 2** | GACACAATTCGAGATCGC | CTTTTCGATCACTCCGTC |
| **High CpG / Low G+C 3** | GTTGAATTGTTGAATATTAT | ATTATAAAAATACATAAACAC |
| **High CpG / Medium G+C** | GAGATGTTATTTTATTGTATTGT | ATACTTCTCAAACCTTTAC |
| **IAP elements** | TTGATAGTTGTGTTTTAAGTGGTAAATAAA | AAAACACCACAAACCAAAATCTTCTAC |
